# Experimental flows through an array of emerged or slightly submerged square cylinders over a rough bed

**DOI:** 10.1038/s41597-020-00791-w

**Published:** 2021-01-11

**Authors:** Marina Oukacine, Sébastien Proust, Frédérique Larrarte, Nicole Goutal

**Affiliations:** 1 Saint-Venant Laboratory for Hydraulics (LHSV), Chatou, France; 2 Riverly Research Unit, INRAE, Villeurbanne, France; 3 Univ. Gustave Eiffel, Marne la Vallée, France; 4 EDF R&D, National Laboratory for Hydraulics and Environment (LNHE), Chatou, France

**Keywords:** Natural hazards, Fluid dynamics

## Abstract

The experimental dataset presented was collected in an 18 m long and 1 m wide laboratory flume. Low to high flood flows through an urbanized floodplain were modelled. The floodplain bed is rough, modelled with dense artificial grass. A square cylinder array, representing house models, was set on the rough bed. The cylinder immersion rate was varied: cylinders are emerged for three flow cases and slightly submerged for one case. The experimental dataset comprises water levels, measured using an ultrasonic transit time probe, velocities across the channel measured using an Acoustic Doppler Velocimetry with a side looking probe, and velocities in longitudinal-vertical planes measured using Particle Image Velocimetry. These data could help understanding the physical processes associated with high flood flows through urbanized floodplains, with a focus on the transition from emerged to submerged obstacles. They could also be used as benchmark data to assess the ability of numerical models from one to three-dimensions to estimate the flood hazard (water depth, velocity) over a wide range of flood event magnitudes.

## Abstract (summary)

**Table:** 

Measurement(s)	velocity • depth of water
Technology Type(s)	particle-image velocimetry • ultrasonic velocimetry • ultrasonic transit time probe
Factor Type(s)	square cylinder immersion rate

Machine-accessible metadata file describing the reported data: 10.6084/m9.figshare.13286915

## Background & Summary

**Fig. 1:**
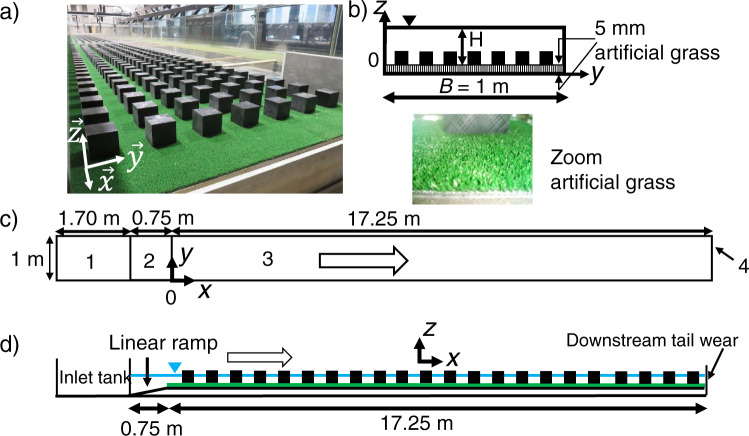
(**a**) Open-channel flume at INRAE Lyon-Villeurbanne (view looking upstream); (**b**) sketch of a cross-section (view looking upstream) with a zoom of the artificial grass; (**c**) sketched top view of the flume without the square cylinders: 1. Inlet tank, 2. Vertical linear ramp with a 15% slope rising the water until the synthetic grass bed level, the water level is nearly flat in the upstream part of the flume, 3. Working channel length, 4. Downstream tail weir; (**d**) sketch of the lateral view of the entire flume.

As a result of climate change, extreme floods will become more frequent and more intense. People and properties, such as housing and industrial facilities, must therefore be protected against these floods for which data are very scarce or even non-existent^1^. When moving from low to extreme flooding, the flood extent over the floodplain greatly varies. This study aimed at investigating if there is a change in the physical processes when the houses initially emerged become weakly submerged and at providing well documented data for validation of simulations. To our knowledge, very few experimental studies have focused on this issue by considering a dense urban area, as depicted in Fig. 1a^2^. Experimental study exists^3–10^ and used blocks up to 25. Most of them provide only water level measurements and are not interested in the study of the vertical confinement. Another complementary area of research concerns the development of simulation tools for extreme flows in a congested area. The field data are very scarce so the experimental data are essential to validate the different ways of modellisation. One-dimensional models are reasonably convenient to simulate flood propagation in straight streets, except near-street intersections where the flow is typically highly perturbed, and strongly 2-D or even 3-D^11^. 2-D modelling was improved by verifying the accuracy of the roughness approach against full buildings incorporation in flood simulation^12^ or by representing the urban area topography^13^ or by introducing porosity in the model^14–17^ in order to take into account the building blockage effect^7,18^. Recently, more information can be found in a review of the data available^19^.

As extreme flood data are very scarce or even non-existent in the field, the present dataset could help understanding the physical processes associated with extreme flood flows in a large simplified urban district area. Moreover, these data could be used as benchmark data to assess the ability of numerical models from one-dimension (1D) to three-dimensions (3D) to estimate the flood hazard over a wide range of flood event magnitudes^2,20^.

The experiments were carried out in an 18 m long and 1 m wide open-channel flume (Fig. 1a), which is located in the Hydraulic and Hydro-morphology Laboratory (HHLab) at INRAE Lyon-Villeurbanne, France. The channel bed slope in the longitudinal direction, *S*_0_, is equal to 1.05 mm/m, and the working length is 17.25 m (Fig. 1c). The cross-section is rectangular (Fig. 1b) with the right-hand sidewall made of glass, and the left-hand sidewall (removable) made of plexiglass. The channel bed is covered with dense artificial grass (5 mm high rigid blades with a density of 256 blades per square centimeter), see Fig. 1a,b. Over the artificial grass, 833 cylinders were placed with an in-line distribution (Fig. 1a). Each cylinder has a square section (side length $\ell =6.4$ cm) and has a height *k* = 5.92 cm when measured from the average top of the grass blades. The distance between two adjacent square cylinders is constant, the same in the transverse and longitudinal directions, and equals to 7.9 cm (Fig. 1a). The distance between the sidewall and the closest square cylinder is equal to 3.9 cm. The most upstream transverse square cylinder row is located at longitudinal position *x* = 0.17 m (see the origin of *x* in Fig. 1a). The array comprises 119 transverse rows of square cylinders, with 7 square cylinders in each row. At the downstream end of the flume (*x* = 17.25 m), an adjustable vertical weir enables the water surface to be controlled.

This physical model is at scale 1/100. At real scale, the spacing between two adjacent houses, $L-\ell$, (where *L* is the distance between two square cylinder centres) corresponds to a two-way motorway including sidewalks, and the height of the house, *k*, corresponds to that of a single-storey house (approximately 6 m high). The aligned configuration was chosen to simplified a suburban area. Note that, because of the presence of the square cylinders, the classical bed friction laws are not applicable to these highly confined flows with small immersion rates.

Four streamwise uniform flows on average were investigated, varying the immersion rate *H*/*k*, where *H* is water depth and *k* is square cylinder height, with *H*/*k* = 0.42; 0.93; 0.98; 1.48. The hydraulic parameters are reported in Table 1, in which $F{r}_{{U}_{Q}}(H)={U}_{Q}/\sqrt{gH}$ is the Froude number, $R{e}_{{U}_{Q}}(H)={U}_{Q}H/\nu$ is the Reynolds number, ${U}_{Q}=Q/A$ is bulk velocity, *A* = *BH* being the wet area and *B* the channel width. Note that, the data with immersion rate *H*/*k* = 0.93 and 1.48 are more exhaustive than the other two flow cases because the velocity measurement is more detailed (see section Methods subsection ADV and PIV velocity measurements positions).

**Table 1:** Flow parameters of the test cases: *H* is water depth, *k* is square cylinder height, *Q* is flow rate, *U*_*Q*_ is bulk velocity, *H*_*weir*_ is the height of the downstream weir, *λ*_*f*_ is frontal density per unit horizontal surface, $F{r}_{{U}_{Q}}(H)$ is the Froude number and $R{e}_{{U}_{Q}}(H)$ is the Reynolds number.

N° [—]	*H*/*k* [—]	*H* [m]	*Q* [m^3^.s^−1^]	*U*_*Q*_ [m.s^−1^]	*H*_*weir*_ [m]	*λ*_*f*_ [—]	${\boldsymbol{F}}{{\boldsymbol{r}}}_{{{\boldsymbol{U}}}_{{\boldsymbol{Q}}}}{\boldsymbol{(}}{\boldsymbol{H}}{\boldsymbol{)}}$ [—]	${\boldsymbol{R}}{{\boldsymbol{e}}}_{{{\boldsymbol{U}}}_{{\boldsymbol{Q}}}}{\boldsymbol{(}}{\boldsymbol{H}}{\boldsymbol{)}}$ [—]
1	0.42	0.025	0.0016	0.064	0.013	0.08	0.130	1590
2	0.93	0.055	0.0033	0.060	0.037	0.17	0.082	3280
3	0.98	0.058	0.0038	0.065	0.038	0.18	0.086	3770
4	1.48	0.088	0.0103	0.117	0.059	0.185	0.120	101000

## Methods

A right-handed Cartesian coordinate system was used (Fig. 1), in which the *x*-axis is aligned with the longitudinal direction, parallel to the flume bottom, the *y*-axis is aligned with the spanwise direction, and the vertical *z*-axis is aligned with the vertical direction (normal to channel bed). The origin *x* = 0 is located at the beginning of working length (Fig. 1c), *y* = 0 at the right-hand sidewall, and *z* = 0 at the top of the grass blades (Fig. 1b).

### Determining the flow rate

The values of immersion rate *H*/*k* and water depth *H* were chosen in collaboration with the other partners of the FlowRes project^21^ (https://flowres.inrae.fr/en/), with experiments in four laboratories. *H*/*k*-values are thus input data while flow rate (*Q*) values are data to be determined.

The inlet flow rate is controlled by a control valve Samson 3310 with servomotor PSQ, and is monitored with an electromagnetic flow meter (Krohne Waterux 3000 IFC 100). The discharge measurement uncertainty is 0.3% of the maximum range set either here ± 0.16 L.s ^−1^, calculated with manufacturer data for a maximum flow rate of 50 L.s^−1^. For the four flows, the flow rate was found after successive iterations of the couple ‘discharge/height’ of the downstream weir. More details can be found in estimating the flow rate with presence of obstacle in^22^.

### Determining the square cylinder height

**Fig. 2:**
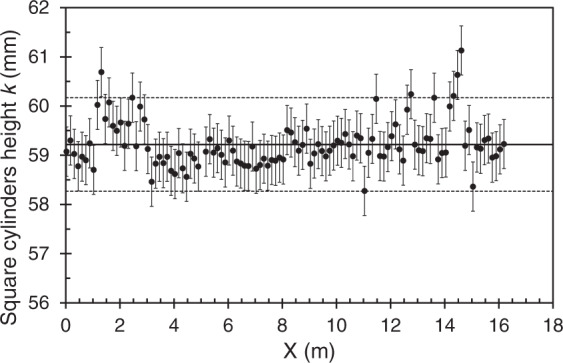
Average height of the square cylinders in each transverse row measured from the top of the grass blades at various longitudinal positions. The solid line indicates the spatial average in the horizontal plane $< k(x,y){ > }_{i,j}\,=0.0592$ m and the dotted lines represent ±2 *σ*(*x*, *y*), where *σ*(*x*, *y*) is the standard deviation of the local average height *k*(*x*, *y*).

At the flume bottom, the artificial grass was glued to 1 cm thick PVC sheets, which in turn were glued to the glass bottom of the flume. The square cylinders were then screwed to the PVC sheets. Each cylinder partly crushed the grass at its location. Considering that the flow within the dense artificial grass is negligible (256 blades per square centimetre with 1 mm wide blades), the vertical distance from the cylinder top to the top of blades was to be determined. Measurements of this distance were made at 6 positions along the *y* axis for 230 positions along the *x* axis, i.e. a total of 1380 positions. 115 positions along the *x* axis correspond to the centres of cylinder rows (except for 4 rows that are not reachable by the measuring devices set on the traversing system). 115 other positions along the *x* axis correspond to the centres of rows without cylinders. The difference between the air depth measurement at the cylinder top and that taken at the bed level (dense meadow) is the cylinder height. We obtained a spatially averaged height $< k(x,y){ > }_{x,y}\,=0.0592$ m as shown in Fig. 2, with *i* the index along the streamwise direction and *j* along the spanwise direction. This value is obtained using the following equation:

1 $$ < k(x,y){ > }_{i,j}=\frac{1}{115}\mathop{\sum }\limits_{i=1}^{115}\frac{1}{6}\mathop{\sum }\limits_{j=1}^{6}k(x,y)$$

where *k*(*x*, *y*) is the average height at position (*x*, *y*)

### Determining the water depth

**Fig. 3:**
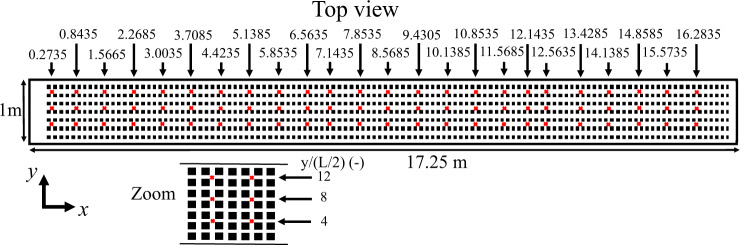
Positions (red markers) of the flow depth measurements using the ultrasonic probe. Top: global top view. Bottom: zoomed section indicating the positions along the *y* axis of the three longitudinal transects.

The average water depth is defined as the difference of air depths with and without water (both measured using the ultrasonic probe). The average water depth *H*(*x*, *y*) was measured at 24 positions along the *x* axis and at 3 lateral positions *y*/(L/2) = 4,8 and 12 as shown in Fig. 3.

The spatial average of the water depth $$ < H(x,y){ > }_{x,y}$$ will be merely noted as *H*, with *i* the index along the streamwise direction and *j* along the spanwise direction, calculated as follows:

2 $$H=\frac{1}{24}\mathop{\sum }\limits_{i=1}^{24}\frac{j}{3}\mathop{\sum }\limits_{y=1}^{3}H(x,y)$$

The convergence time of the time-averaged water depth was estimated at two different locations, in a central vein, and behind an obstacle model, where the vertical boundary layer was assumed to be established at 12 m. The convergence time is the time needed for the value to be stationary and was determined when the relative deviation tends toward zero. For each flow case, water depth is measured independently for a duration of *t* = 10, 20, 30, 40, 60, 90 and 100 s. The convergence time found for each flow case is equal to 40 s. Once the convergence time has been established, the measurement is then carried out. It is ensured that all measurements are within the interval defined as 2*σ* with *σ* the standard deviation of the mean value *H*(*x*, *y*). Obtaining a constant water level flow flushing the obstacle tops is difficult because of the variation in the local cylinder height *k*(*x*, *y*) (Fig. 2). The average water depth at *x* = 12 m (study area) for case n°3 is *H* = 58.3 mm.

### Determining the velocity

Velocity measurements were performed in the central vein at *y*/(*L*/2) = 6 and at positions *x* = 4, 8, 12 and 15 m to investigate the longitudinal flow development. The emerged obstacles flows *H*/*k* = 0.42, 0.93, 0.98 are developped from 12 m and the submerged obstacle flow *H*/*k* = 1.48 is developped from 8 m. This is determined when the vertical profiles of average velocity overlap.

**Fig. 4:**
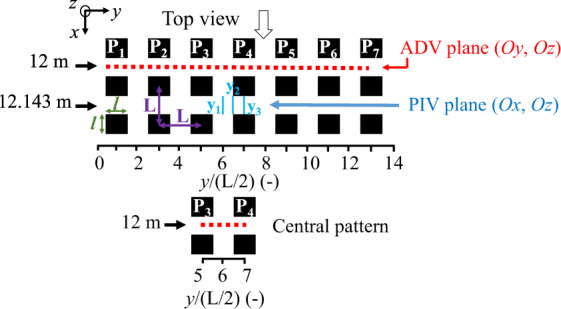
Positions of the PIV measurement *y*_1_, *y*_2_ and *y*_3_, and ADV measurements across the channel and central pattern; with *L* = 14.3 cm, $\ell$ = 6.4 cm and $L-\ell$ = 7.9 cm for the 4 flows.

PIV measurements were performed in the vertical-longitudinal plane while ADV measurements were performed across the channel (vertical-lateral plane). They thus complement each other to explore the physical processes involved and can be compared. The convergence time of the time-averaged velocities and turbulence statistics was estimated for the ADV data at positions *y*/(*L*/2) = 6; 6.55; 7 (Table 2), and at 3 elevations *z*: near the channel bed, at *z*/*H* = 0.40 and near the free surface (Table 3). For the emerged obstacles flows *H*/*k* = 0.42, 0.93, 0.98, the convergence time is *t* = 400 s in the central vein and near the obstacle edge and *t* = 300 s behind the obstacle. For the submerged obstacle flow *H*/*k* = 1.48, the convergence time *t* = 300 s everywhere. In these experiments an elementary pattern extends from the middle of the prism *P*_3_ to the middle of the prism *P*_4_ (Fig. 4) and is located between 5 ≤ *y*/(*L*/2) ≤ 7. This elementary pattern will be termed central pattern.

**Table 2:** Summary of the duration *t* for the convergence of time-averaged velocity and turbulence statistics for flow cases with *H*/*k* = 93% and 148% Measurements for elementary pattern located in the central vein at *y*/(L/2) = 6, at the edge of the square cylinder at *y*/(L/2) = 6.55 and behind the middle of the obstacle *y*/(L/2) = 7 .

*H*/*k* [%]	Convergence time t [s]		
	Pattern: central vein		
	*y*/(*L*/2) = 6	*y*/(*L*/2) = 6.55	*y*/(*L*/2) = 7
93	400	400	300
148	300	300	300

In the same open-channel flume, with only artificial grass over the bed (no house models) and higher flow rates, the experimental work of ^23^ showed duration *t* for the convergence of time-averaged velocity and turbulence statistics around 120 s.

### Measurement of the water level fluctuations

Free surface oscillations measurements were carried out along and across the flume with the ultrasonic transit time probe. Two types of measurements were carried out, the first one is a measure across the flume of *t* = 200 s for each position with a total of 35 positions and the same measure was made 5 hours after the first measured position, the second one is a measure of 12 hours at the position *x* = 12 m and *y*/(L/2) = 13. These oscillations are mainly in the transverse direction and are caused by the vortex shedding behind the obstacles, and are termed seiching phenomenon. Seiching is the most important for the flow with *H*/*k* = 0.42 with a maximum standard deviation of the level fluctuations equal to 1.5% in the centre of the channel for measurements taken at a time *t* = 0 h and after 5 hours. All flows have the same oscillation mode but the higher the water height, the lower the oscillation. The water level fluctuations stabilize after various durations depending on the flow cases: after 4 hours for *H*/*k* = 0.42, 1 hour for *H*/*k* = 0.93 and 0.98 and after 3 hours for *H*/*k* = 1.48. For more details about seiching phenomenon due to vortex shedding in the flow through cylinder arrays see^24–27^ in this open-channel flume in particular, see the experiments of ^28^.

### ADV and PIV velocity measurements positions

The positions of the velocity measurements using PIV and ADV are summarized in Fig. 4. The PIV measuring planes for all flow cases are located at lateral positions *y*_1_/(*L*/2) = 6, *y*_2_/(*L*/2) = 6.55 and *y*_3_/(*L*/2) = 7 and at *x* = 12.143 m.

ADV measurements were carried out at *x* = 12 m, with 1 cm spacing the spanwise direction between two measurements across almost the entire width of the channel (Table 3), and at various elevations.

**Table 3:** Elevations of the ADV velocity measurements across the channel for the four flows studied with *H*/*k* = 42%, 93%, 98% and 148%.

ADV Vertical measurement elevations in the transverse plan (*Oy*, *Oz*) at *x* = 12 m		
**42% immersion**		
*z* [mm]	*z*/*H* [−]	*z*/*k* [−]
11	0.44	0.186
**93% immersion**		
*z* [mm]	*z*/*H* [−]	*z*/*k* [−]
11	0.20	0.186
24.2	0.44	0.41
35.2	0.64	0.60
**98% immersion**		
*z* [mm]	*z*/*H* [−]	*z*/*k* [−]
11	0.19	0.186
25.6	0.44	0.43
**148% immersion**		
*z* [mm]	*z*/*H* [−]	*z*/*k* [−]
11	0.125	0.186
36.96	0.42	0.62
58.96	0.67	1.00
79.33	0.90	1.34

The central pattern, from *y*/(*L*/2) = 5 to 7, is indeed a large pattern (Fig. 4), as it corresponds to two patterns in the literature. Usually, in the literature a pattern ranges from *y*/(*L*/2) = 5 to 6 or 6 to 7 corresponding to half a vein and half an obstacle. An elementary pattern in the literature includes half a prism and half a central vein. Our elementary pattern is twice wider to verify the symmetry usually assumed in a central vein. In addition, the fact that a longitudinal row of square cylinders is present in the middle of the channel, i.e. in its privileged central vein (for a channel without obstacles), prevents certainty on the symmetry hypothesis.

**Fig. 5:**
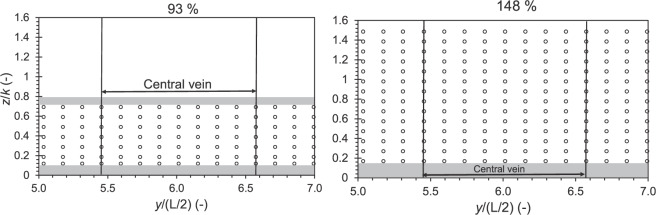
ADV measuring mesh realized within for the central pattern (5 ≤ *y*/(*L*/2) ≤ 7) for cases (left) *H*/*k* = 93% and (right) 148%, the grey areas indicate the blind zones for measurements.

At the position, *x* = 12 m, an ADV punctual velocity profiles are measured with a spanwise spacing of 1 cm between two profiles at the positions 5 ≤ *y*/(*L*/2) ≤ 7, 15 positions along the *y* axis between the centre of the prism *P*_3_ and the centre of the prism *P*_4_ with 8 positions along the *z* axis for *H*/*k* = 93% and 15 positions along the *z* axis for *H*/*k* = 148% (Fig. 5). The measuring volume of the ADV has a cylindrical shape of 6 mm diameter and 7 mm length.

## Data Records

The experimental data have been uploaded on the Zenodo website^29^, under the DOI number 10.5281/zenodo.3871773. Four Excel files termed according to the value of immersion rate *H*/*k* can be found. In each files, several sheets present the water depth, the ADV time-averaged velocities and turbulence statistics across the channel at *x* = 12 m (described in Table 3), the ADV velocities at *y* = 6 for 4 different positions *x* = 4,8,12,16 m. The last sheet describes the central pattern ADV velocities and turbulence statistics for the two flows with *H*/*k* = 93% and 148% flows.

## Technical Validation

### Flow sensor and displacement system

The inlet flow rate is controlled by a control valve Samson 3310 with servomotor PSQ, and is monitored with an electromagnetic flow meter (Krohne Waterux 3000 IFC 100). The discharge measurement uncertainty is 0.3% of the maximum range set either here ± 0.16 L.s^−1^, calculated with manufacturer data for a maximum flow rate of 50 L.s^−1^.The flow sensor was used throughout the measurement campaign showing that the flow rate used for a given water level was replicated and reproducible within the device’s margin of error.

The channel is equipped with a motorized displacement system (Siemens S7-1200), carrying the measuring devices. The accuracy along the three space directions is of 0.1 mm. These measuring devices are an ultrasonic probe with transit time and an Acoustic Doppler Velocimetry (ADV) with a 3D side looking probe.

Note that the laboratory ambient air temperature is maintained by a thermostat between 19 °C and 23 °C.

### Water level

The ultrasonic probe with transit time measures the distance between the probe and the first reflecting surface. It is used between the transmitter and the free surface and also between the transmitter and the bottom of the channel without water. This difference gives the water depth. This apparatus is manufactured by Baumer (UNDK 20I6903/S35A). Accuracy is better than 0.3 mm and the reproducibility is better than 0.5 mm according to the manufacturer. The chosen acquisition rate is 50 Hz (maximum value permitted). The height of water determined for a flow was tested during the entire measurement campaign. Replicability was therefore verified.

### Velocity measurements

The ADV velocity probe chosen acquisition rate is 100 Hz for a given acquisition duration. The measurement uncertainty on velocity is ±0.5% of the measured value ±1% mm.s^−1^ (manufacturer data). The centre of the measuring volume is located at 5 cm away from the probe transmitter.

For all the flows studied, the ADV measurement volumes are independent, with vertical intervals of 6 mm. It is essential to ensure that the ADV probe is orthogonal to the planes of space. The probe verticality is obtained by using a plumb line. The axis *Oz* perpendicular to the bottom of the channel is therefore confused with the vertical. Since the exact alignment of the ADV probe with the transverse axis is difficult to obtain^30^, a first test consists in placing the measurement volume near the wall and rotating the probe along the vertical axis in order to cancel the transverse mean velocity component. The second step consists of post-processing the velocity measurements assuming that the vertical average of the transverse component of the time-averaged velocity is nil at the wall. Hence, a rotation angle is deduced to ensure that the depth-averaged transverse velocity over the water level is zero. This rotation angle should be lower than 1 degree according to^30^.

This rotation angle *θ*_*z*_ is applied to all lateral and longitudinal time-averaged velocity values as a function of the longitudinal position *x*, because this angle also depends on the verticality of the wall. The Table 4 summarizes the variation of the angle *θ*_*z*_ according to the position *x*. The longitudinal position *x* = 12 m has 3 different angles because the probe has been taken off twice.

**Table 4:** Rotation angle *θ*_*z*_ around the vertical axis of the ADV probe as a function of the position along the *x* axis.

Position *x* [m]	Angle *θ*_*z*_ [°C]
4	0.073
8	−0.078
12	−0.0627
12	0.230
12	−0.220
15	−0.715

The particules used were polyamide powder of 50 *μ*m median diameter (Evonik Vestosint 1164 white). They were used to seeding the flow during the ADV measurements. It enables to increase the signal to noise ratio and the correlations inside the measuring volume. For some ADV measurements, hollow glass particles (Hologlass sphere) of 10 *μ*m diameter were also used. The inlet tank has not been cleaned up because the mixture of particles is not problematic for ADV measurements whereas it has been for PIV measurements. Indeed, for PIV measurements, two different particle sizes imply that the illumination will not be homogeneous. Only hollow glass particules were used for the PIV measurements.

**Fig. 6:**
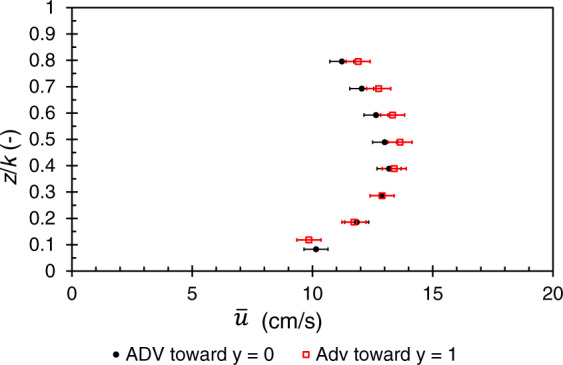
Vertical profile of the longitudinal time-averaged velocity ū at *x* = 12 m and *y*/(*L*/2) = 6, towards the glass sidewall located at *y* = 0, or towards the plexiglass sidewall positioned at *y* = 1; after taking into account the correction alignment angle of the probe (Table 4) for the flow H/k = 0.93.

The Fig. 6 shows that with a different rotation angle correction *θ*_*z*_ (Table 4) and less than 1° ^30^, it is possible at the same point to obtain at the measurement uncertainty almost the same velocity profile whatever the direction of the probe (towards *y* = 1 or *y* = 0).

As regards the PIV measurements, the artificial grass was cut so that the laser could go through it, so some perturbations are expected, and also we made sure that the laser sheet was straight and the calibration with the camera at a very low angle. The Table 5 presents the frequency and the time between two image bursts used for each flow case.

**Table 5:** Frequencies and time between two image burst used for the PIV measurements as a function of the lateral position *y* for each flow case.

*H*/*k* [%]	Frequencies [Hz]		
	*y*/(*L*/2) = 6	*y*/(*L*/2) = 6.55	*y*/(*L*/2) = 7
42	4	3	3.5
93	5	3	3.5
98	5	3	3.5
148	2.5	3	4
	**Time between two image burst [*μ*s]**		
***H*/*k*** **[%]**	***y*/(*L*/2) = 6**	***y*/(*L*/2) = 6.55**	***y*/(*L*/2) = 7**
42	4000	8000	—
93	1000	20000	16500
98	1000	8000	16500
148	1000	6000	9000

### Data usage caution

It should be noted that aligning 833 square cylinders was not an easy task. We estimated a maximum shift of 5 mm in both longitudinal and transverse directions which represents 8% of the square cylinder width.

## Usage Notes

This dataset is intended to bring insight into the transition from emergence to slight submergence of house models focusing on their impact on the velocity field in a dense urban area. As extreme flood data are very scarce or non-existent in the field, this dataset could be used as a benchmark data for other experimental studies and numerical modelling of flows through a simplified urban area. This is relevant in the flood risk management.

Another use of this dataset is to compare the data with other configurations and to conclude upon the physical processes involved.

## Data Availability

WinADV software was used to process the ADV velocity data. Data were filtered using the despiking technique of ^31^. The data measured with the PIV system were post-processed with Davis software from LaVision company.

## References

[1] European Union. Directive 2007/60/ce du parlement européen et du conseil, du 23 octobre 2007, relative à l’évaluation et à la gestion des risques d’inondation. *Official journal of the European Union* 27–34 (2007).

[2] Oukacine, M. Etude expérimentale et numérique des écoulements à surface libre en présence d’obstacles émergés et faiblement submergés. *PhD Thesis, Université Paris-Est* (2019).

[3] Herbich JB, Shulits S (1964). Large-scale roughness in open-channel flow. Journal of the Hydraulics Division.

[4] Testa G, Zuccalà D, Alcrudo F, Mulet J, Soares-Frazão S (2007). Flash flood flow experiment in a simplified urban district. J. Hydraul. Res..

[5] LaRocque LA, Elkholy M, Chaudhry MH, Imran J (2013). Experiments on urban flooding caused by a levee breach. J. Hydraul. Eng..

[6] Huang C-J, Hsu M-H, Teng W-H, Wang Y-H (2014). The impact of building coverage in the metropolitan area on the flow calculation. Water.

[7] Zhou Q, Yu W, Chen AS, Jiang C, Fu G (2016). Experimental assessment of building blockage effects in a simplified urban district. Procedia Engineering.

[8] Velickovic M, Zech Y, Soares-Frazão S (2017). Steady-flow experiments in urban areas and anisotropic porosity model. Journal of Hydraulic Research.

[9] Finaud-Guyot P, Garambois PA, Dellinger G, Lawniczak F, François P (2019). Experimental characterization of various scale hydraulic signatures in a flooded branched street network. Urban Water Journal.

[10] Li X (2019). Laboratory modelling of urban flooding: strengths and challenges of distorted scale models. Hydrol. Earth Syst. Sci..

[11] Neary VS, Sotiropoulos F, Odgaard AJ (1999). Three-dimensional numerical model of lateral-intake inflows. J. Hydraul. Eng..

[12] Beretta R, Ravazzani G, Maiorano C, Mancini M (2018). Simulating the influence of buildings on flood inundation in urban areas. Geosciences.

[13] El Kadi Abderrezzak K, Paquier A, Mignot E (2009). Modelling flash flood propagation in urban areas using a two-dimensional numerical model. Nat. Hazards.

[14] Guinot V, Soares-Frazão S (2006). Flux and source term discretization in two dimensional shallow water models with porosity on unstructured grids. Int. J. Numer. Methods Fluids.

[15] Guinot V (2012). Multiple porosity shallow water models for macroscopic modelling of urban floods. Adv. Water Resour..

[16] Viero DP (2019). Modelling urban floods using a finite element staggered scheme with an anisotropic dual porosity model. J. Hydrol..

[17] Ferrari A, Viero DP (2020). Floodwater pathways in urban areas: A method to compute porosity fields for anisotropic subgrid models in differential form. J. Hydrol..

[18] Turki S, Abbassi H, Nasrallah S (2003). Effect of the blockage ratio on the flow in a channel with a built-in square cylinder. Computational Mechanics.

[19] Mignot E, Li X, Dewals B (2019). Experimental modelling of urban flooding: A review. J. Hydrol..

[20] Oukacine, M. *et al*. Large Eddy Simulation for flows through emerged or slightly submerged square cylinders. In *Proc. 10th RiverFlow Conference on Fluvial Hydraulics* (Ed. Taylor & Francis.) 8 (Delft, 2020).

[21] Proust, S. *et al*. Predicting the flow on the flooplains with evolving land occupations during extreme flood events (FlowRes ANR project). In *Proc. 3rd European Conference on Flood Risk Management FLOODrisk* (Ed. EDP Sciences) 04004 (2016).

[22] Guillen-Ludena S, Lopez D, Mignot E, Rivière N (2020). Flow resistance for varying density of obstacles on smooth and rough bed. Journal of Hydraulic Engineering.

[23] Dupuis, V. Experimental investigation of flows subjected to a longitudinal transition in hydraulic roughness in single and compound channels. *PhD thesis Université Claude Bernard Lyon* 1 (2016).

[24] Zima L, Ackermann NL (2002). Wave generation in open channels by vortex shedding from channel obstructions. Journal of Hydraulic Engineering.

[25] Defina A, Pradella I (2014). Vortex-induced cross-flow seiching in cylinder arrays. Advances in Water Resources.

[26] Zhao K, Cheng N-S, Huang Z (2014). Experimental study of free-surface fluctuations in open-channel flow in the presence of periodic cylinder arrays. Journal of Hydraulic Research.

[27] Viero DP, Pradella I, Defina A (2017). Free surface waves induced by vortex shedding in cylinder arrays. J. Hydraul. Res..

[28] Chetibi M, Proust S, Benamar S (2020). Transverse surface waves in steady uniform and non-uniform flows through an array of emergent and slightly submerged square cylinders. Journal of Hydraulic Research.

[29] Oukacine M, Proust S, Larrarte F, Goutal N (2020). Zenodo.

[30] Peltier Y (2013). Estimation of the error on the mean velocity and on the Reynolds stress due to a misoriented ADV probe in the horizontal plane: Case of experiments in a compound open-channel. Flow Measurement and Instrumentation.

[31] Goring D-G, Nikora V-I (2002). Despiking acoustic doppler velocimetry data. Journal of Hydraulic Engineering.

